# How does perceived risk mediate associations between perceived safety and parental restriction of adolescents’ physical activity in their neighborhood?

**DOI:** 10.1186/1479-5868-9-57

**Published:** 2012-05-18

**Authors:** Alison Carver, Anna Timperio, Kylie Hesketh, David Crawford

**Affiliations:** 1 Centre for Physical Activity and Nutrition Research, School of Exercise and Nutrition Sciences, Deakin University, Burwood, VIC, Australia

**Keywords:** Constrained behavior, Victimization, Mediation, Youth

## Abstract

**Background:**

There is evidence that adolescence is a critical period of decline in physical activity. However, adolescents may have limited opportunities to be physically active outdoors if their parents are concerned about neighborhood safety and restrict their adolescent’s physical activity within their neighborhood. Pathways that lead to parental restriction of adolescents’ physical activity (constrained behavior) are under-researched. This study aimed to examine perceived risk as a potential mediator of associations between perceived safety/victimization and constrained behavior.

**Methods:**

Cross-sectional study of adolescents (43% boys) aged 15–17 years (n = 270) in Melbourne, Australia. Parents reported perceived safety (road safety, incivilities and personal safety) and prior victimization in their neighborhood, perceived risk of their children being harmed and whether they constrained their adolescent’s physical activity. Constrained behavior was categorized as ‘avoidance’ or ‘defensive’ behavior depending on a whether physical activity was avoided or modified, respectively, due to perceived risk. MacKinnon’s product-of-coefficients test of mediation was used to assess potential mediating pathways between perceived safety/victimization and constrained behavior.

**Results:**

For girls only, perceived risk was a significant mediator of associations between perceived road safety and avoidance/defensive behavior, and between perceived incivilities, perceived personal safety, victimization and defensive behavior.

**Conclusions:**

Associations between perceived safety/victimization and constrained behavior are complex. Findings may guide the design of interventions that aim to improve actual and perceived levels of safety and reduce perceptions of risk. This is of particular importance for adolescent girls among whom low and declining levels of physical activity have been observed worldwide.

## Background

Longitudinal studies have demonstrated that participation in regular physical activity during adolescence is important for the prevention of chronic disease in adulthood [1,2]. For example, in the Young Finns study [1], adolescents who were physically active over a six-year study period exhibited fewer biological risk factors for cardiovascular disease, such as elevated serum lipids and insulin levels, and abdominal adiposity, as well as fewer behavioral risk factors such as smoking and high intake of saturated fat. Furthermore, a US longitudinal study [3] that followed girls aged 9–10 over a period of nine years found that a decline in physical activity during that time was associated with a greater increase in body mass index (BMI) and sum of skin fold thickness.

A key determinant of adolescents’ physical activity levels is the time they spend outdoors [4]. Nowadays, however, young people are subject to greater restrictions on spatial boundaries and enjoy lower levels of independent mobility compared with previous generations [5,6]. In addition, participation rates in active transport (e.g., walking and cycling to school) have declined in England [7], Australia [8] and the USA [9], while rates of car travel to school and other destinations have increased. These declines in physical activity are concerning given the health benefits associated with regular physical activity among youth [1,2]. Many adolescents now have limited opportunities to spend time outdoors due to parental concern about neighborhood safety. Parents are concerned specifically about ‘road safety’ and ‘stranger danger’, and these concerns may cause them to restrict or ‘constrain’ their adolescent’s physical activity and active transport within their neighborhood [10,11].

Constrained behavior refers to the modification of habitual activities due to perceived risk of victimization (e.g. how likely a parent considers their child is to be harmed) [12]. Perceived risk is estimated following cognitive assessment of factors such as perceived safety and prior victimization [12]. Constrained behavior can be categorized as either ‘avoidance behavior’ (i.e. not engaging in an activity at all) or ‘defensive behavior’ (i.e. modification to reduce risk) [12,13]. For example, if a parent considers their child to be at risk of being knocked down while walking/cycling to school, they may choose to drive their child to school instead (avoidance behavior) or to accompany their child while walking/cycling to school (defensive behavior). Safety concerns have been associated with restriction of adults’ physical activity, in particular among women and older adults [14], but few studies have focused on parental restriction of their children’s physical activity. Our previous work established that perceived safety [15] and constrained behavior [13] were associated with physical activity among children and adolescents, and that perceived risk of harm was associated with constrained behavior [13] among adolescents (aged 15–17 years), but not among children (aged 10–11 years). However, the pathways that lead to constrained physical activity behavior have not been fully explored.

This study is among the first to examine whether perceptions of safety/victimization are associated with constrained physical activity behavior and whether this association is mediated or explained by perceptions of risk. Considering that constrained behavior is related to lower physical activity among adolescent girls, in particular [13], understanding pathways that lead parents to constrain adolescents’ behavior is essential. Strategies to increase youth physical activity are unlikely to be successful if parental fears and perceptions of risk are not addressed. The present study aimed to examine perceived risk as a potential mediator of associations between perceived safety/victimization and constrained behavior.

## Methods

### Sample

This cross-sectional analysis includes data from the five-year follow-up of the Children Living in Active Neighborhoods Study (CLAN) [13]. Ethics approval was obtained from the Deakin University Ethics Committee, the Department of Education and Training Victoria and the Catholic Education Office. Baseline sampling and recruitment methods have been previously described [16,17]. In brief, at baseline (2001) children were recruited from 19 state primary schools in ten high and nine low socioeconomic areas of Melbourne, Australia. Active written parental consent on behalf of their child was mandatory. Baseline participants were 919 10–12 year-olds (44% response rate), referred to as ‘adolescents’ at follow-up. In 2006, 326 adolescents (aged 15–17 years, 35% of baseline sample) again provided active consent from parents (and the adolescents also provided active consent) and participated in the five-year follow-up. Recruitment methods for the follow-up are presented elsewhere [18]. Only data from 2006 are presented here.

There were some baseline differences in weight status, maternal socio-demographics and physical activity between those in the follow-up sample and the remainder of the baseline sample [13]. Follow-up participants were less likely to be overweight/obese (OR = 0.60, 95% CI =0.46-0.79; p < 0.001) and their mothers were more likely to work part-time (OR = 1.49, 95% CI =1.10-2.03; p = 0.010) and to be tertiary educated (OR =2.09, 95% CI =1.55 = 2.82; p <0.001) at baseline. In addition, follow-up participants spent 28.8 minutes more on week days (95% CI = 20.0-37.5; p <0.001) and 28.7 minutes more on weekend days (95% CI =18.6-38.8; p <0.001) engaged in moderate-to-vigorous physical activity at baseline.

### Measures

Parents completed a questionnaire at home which included items about perceived safety, victimization, risk and parental restriction of their adolescent’s behavior (constrained behavior).

#### Perceived safety

Using previously published items [15], parents were asked about their perceptions of road safety, incivilities (signs of physical/social disorder) and their adolescent’s personal safety within their local neighborhood, with response options (and assigned values in parenthesis): (−2) ‘Strongly disagree’, (−1) ‘Disagree’, (0) ‘Neither agree/disagree’, (1) ‘Agree’, (2) ‘Strongly Agree’. Three items measured perceptions of road safety: ‘There are major barriers to walking/cycling in my local neighborhood that make it hard for my child to get from place to place (e.g. freeways, major roads)’; ‘There is heavy traffic in our local streets’; ‘Road safety is a concern in our area’. Four items measured perceptions of incivilities: ‘My neighborhood is generally free from litter, rubbish, graffiti’ (this item was reverse-scored); ‘There is a high crime rate in our neighborhood’; ‘I am worried about trouble-makers hanging around my neighborhood’; ‘Stranger danger is a concern of mine’. Five items measured personal safety: ‘It is safe for my child to play or hang out in the street outside our house’; ‘Lots of children play or hang out in our street’; ‘My neighborhood is safe for my child to walk/cycle around the block alone in the daytime’; ‘My child would be safe walking home from a bus- or train stop at night’; I am worried that my child might be assaulted when out alone in our neighborhood’ (reverse-scored).

Scores were computed for perceptions of road safety, incivilities and personal safety by combining response values. Possible value ranges for these scores were from −6 to 6, from −8 to 8 and from −10 to 10, respectively. Each score had high test-retest reliability (ICC > =0.8), and moderate-to-high internal reliability (Cronbach’s α > 0.5) [15].

#### Victimization

An index of victimization was developed for this study, guided by recommendations about the need for specificity in the definition and measurement of particular forms of victimization in the context of daily life [12] and the need for defined time-frames [19]. To measure prior victimization in relation to stranger danger and road safety, each parent was asked whether they themselves (a) had an unwelcome approach by a stranger, (b) were knocked down as a pedestrian or (c) as a cyclist, within their neighborhood in the last year. These items were repeated in reference to their adolescent and again in reference to anyone else they knew. Responses to these nine items were coded as yes (1) or no (0). A victimization score (with possible value range from 0 to 9) was then computed. Test-retest reliability of this score was high (ICC = 0.8). Due to skewness, the victimization score was dichotomized with values: 0 ‘no prior victimization’; ≥1 ‘some prior victimization’.

#### Perceived risk

Perceived risk was measured by asking parents to respond to three questions about the likelihood of their adolescent being (a) approached by a stranger, (b) knocked down as a pedestrian or (c) knocked down as a cyclist within their neighborhood in the coming year [13]. Response options and coding (in parenthesis) were: (−2) Highly unlikely; (−1) Unlikely; (0) Neither/Don’t know/Doesn’t apply; (1) Likely; (2) Highly likely. Responses were summed to compute the score for perceived risk (possible range −6 to 6, alpha 0.80). Test-retest reliability of this score was moderate (ICC = 0.5).

#### Constrained behavior

Indices of avoidance/defensive behavior in relation to adolescents’ physical activity were developed based on Ferraro’s indices of constrained behavior [12]. To assess avoidance behavior, parents responded to seven statements about preventing their adolescent from doing the following in their neighborhood: (1) playing alone outdoors; (2) playing with friends outdoors; (3) spending time outside after dark; (4) walking/cycling on the street after dark; (5) playing alone on local streets; (6) playing with friends on local streets; (7) walking/cycling with friends [13]. To assess defensive behavior, parents responded to a further seven statements regarding: (1) the need for their adolescent to be supervised when playing outside; preventing their adolescent from doing the following unless supervised: (2) playing outdoors, (3) playing on local streets, (4) walking/cycling in their neighborhood; whether their adolescent (5) has self-defense skills, (6) carries a whistle/alarm to ward of unwelcome strangers; (7) whether the parent is always reminding their adolescent about road safety [13].

Response options and subsequent coding (in parenthesis) were: (−2) Strongly disagree; (−1) Disagree; (0) Neither/Don’t know; (1) Agree, (2) Strongly Agree. In cases where there was a missing value among the score’s component variables, this was replaced with the median value of all other components. Responses were summed to compute scores for avoidance (alpha 0.79) and defensive (alpha 0.62) behavior respectively, each with possible value range of −14 to 14 (a higher score indicated a higher level of avoidance/defensive behavior). The scores for avoidance (ICC = 0.8) and defensive behavior (ICC = 0.9) had high test-retest reliability.

### Data analyses

**Figure 1:**
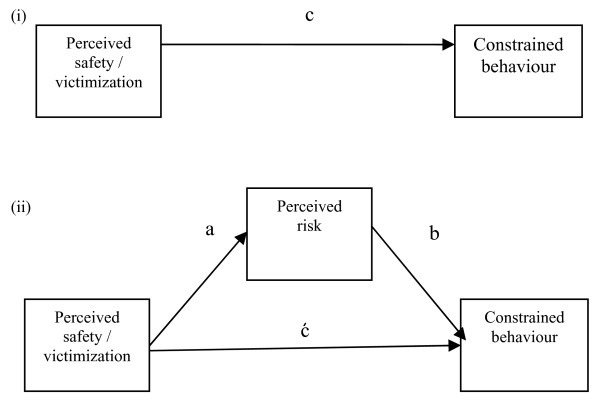
**Path diagrams for total effect and mediation model**. **(I)** the total effect of the independent variable on the dependent variable. **(II)** the indirect effect of the independent variable on the dependent variable through the mediator variable.

Independent sample t-tests were performed to examine differences in perceived safety, perceived risk, avoidance behavior and defensive behavior according to sex of the adolescent. Linear regression analyses, stratified by sex, were performed to examine associations between perceived safety (road safety, incivilities, personal safety) or victimization and constrained (avoidance, defensive) behavior. Using MacKinnon’s product-of-coefficients test of mediation [20], perceived risk was examined as a potential mediator of the above associations. Paths diagrams for (i) the regression and (ii) mediation models are depicted in Figure 1. For perceived risk to be considered a mediator, the following conditions were necessary:

a) a significant association between the perceived safety/victimization score and perceived risk (path a);

b) a significant association between perceived risk and the constrained behavior score, controlling for the perceived safety/victimization score (path b).

Where these conditions were satisfied, the mediated effect was calculated using the product-of-coefficients method (a*b). In addition, to test significance, the mediated effect was divided by its standard error, resulting in a z-score, z_ab._ A magnitude of z_ab_ of greater than 1.96 indicated that the mediated effect was significant (p < 0.05) [20]. In cases where paths a and b were significant (p < 0.05), the proportion mediated was calculated by dividing the mediated effect by the total effect (path c), expressed as a percentage.

## Results

Data were analyzed for 270 adolescents (43% boys) with mean age 16.3 (SD 0.6) years, who had complete data. Most parents who responded were mothers (87%), were married (78%) and almost half (48%) were tertiary educated. Most were employed full-time (37%) or part-time (43%).

### Perceived safety, victimization, perceived risk and constrained behavior

Scores for perceived safety, perceived risk and constrained behavior were distributed normally. Mean values (and standard deviations) for these scores are presented in Table 1. Scores for victimization (not tabulated) had a positively skewed distribution. When scores for boys and girls were examined separately, the median was 0 (indicating no prior victimization) with a range of 0–3 for boys and for girls. There were no significant differences for any of the above scores between parents of boys and girls. A quarter of all parents (25%) reported prior victimization (i.e., their victimization score was greater than one).

**Table 1:** Mean scores (and standard deviations) for perceived safety, perceived risk and constrained behavior

**Scores**	**Mean (SD) score**	
	**Boys (n = 115)**	**Girls (n = 155)**
Perceived Safety		
Road safety^a^	− 1.0 (2.5)	− 1.1 (2.2)
Incivilities^b^	− 1.8 (2.6)	− 2.0 (2.3)
Personal safety^c^	1.7 (2.8)	1.8 (2.9)
Perceived risk^d^	−2.8 (2.2)	−3.2 (2.1)
Constrained behavior	−5.6 (4.2)	−4.4 (5.2)
Avoidance ^e^		
Defensive^f^	−6.6 (3.4)	−6.3 (3.4)

a Range of possible values was −6 to 6. High scores indicate high levels of concern about road safety.

b Range of possible values was −8 to 8. High scores indicate high levels of concern about incivilities.

c Range of possible values was −10 to 10. High scores indicate that levels of personal safety are perceived to be high.

Low scores indicate high levels of concern about adolescent’s personal safety.

d Range of possible values was −6 to 6. Lower scores indicate lower levels of perceived risk.

e Range of possible values was −14 to 14. Lower scores indicate lower levels of avoidance behavior.

f Range of possible values was −14 to 14. Lower scores indicate lower levels of defensive behavior.

### Associations between perceived safety/victimization and constrained behavior

Associations between perceived safety/victimization and constrained behavior for boys and girls are presented in Table 2 (path c). For all participants, greater parental concern about road safety (i.e. higher road safety score) was associated with higher levels of constrained behavior, except for defensive behavior with respect to girls. In addition, greater concern about incivilities was associated with higher levels of constrained behavior, except for avoidance behavior with respect to boys. For all participants, higher levels of perceived personal safety were associated with lower levels of constrained behavior. Victimization was not significantly associated with either type of constrained behavior for boys or girls.

**Table 2:** Examining perceived risk as a mediator of associations between perceived safety/victimization and constrained behavior

		**Avoidance Behavior**						**Defensive Behavior**						
	**c**	**Path Coefficients**		**c**^**/**^	**Mediated Effect**	**Proportion Mediated**	**z**_**ab**_	**c**	**Path Coefficients**		**c**^**/**^	**Mediated Effect**	**Proportion Mediated**	**z**_**ab**_
		**a**	**b**		**ab**	**(%)**			**a**	**b**		**ab**	**(%)**	
**Boys**														
Perceived safety														
Road safety	**0.453**	**0.186**	0.290	**0.399**	**-**	**-**	**-**	**0.329**	**0.186**	0.303	0.272	0.056	1700	1.32
Incivilities	0.246	**0.347**	0.321	0.135	**-**	**-**	**-**	**0.297**	**0.347**	0.266	0.205	-	-	-
Personal	**−0.586**	**0.325**	0.073	**−0.563**	**-**	**-**	**-**	**−0.354**	**−0.325**	0.210	−0.285	-	-	-
Victimization	0.606	**1. 224**	**0.382**	0.137	0.468	77.2	1.36	0.567	**1.224**	**0.365**	0.121	0.447	78.8	1.54
**Girls**														
Perceived safety														
Road safety	**0.389**	**0.201**	**0.708**	0.247	**0.143**	36.8	**2.20**	0.189	**0.201**	**0.559**	0.077	**0.113**	59.8	**2.12**
Incivilities	**0.938**	**0.322**	**0.432**	**0.799**	0.139	**14.8**	1.64	**0.579**	**0.322**	**0.388**	**0.454**	**0.125**	21.6	**2.08**
Personal	**−0.753**	**−0.322**	0.354	**−0.639**	**-**	**-**	**-**	−**0.350**	**−0.322**	**0.444**	−**0.207**	**−0.143**	40.9	**−2.63**
Victimization	1.836	**1.089**	**0.711**	1.062	0.774	42.2	1.89	1.142	**1.089**	**0.550**	0.543	**0.599**	52.5	**2.04**

Path coefficients are: (c), total effect-association between independent variable and dependent variable; (a), association between independent variable and potential mediator; (b), association between potential mediator and dependent variable, adjusted for independent variables; (c^/^), association between independent variable and dependent variable, adjusted for potential mediator. The mediated or in direct effect is denoted by ‘ab’. The z-score ‘Z_ab_’ denotes the mediated effect divided by its standard error. If the magnitude of Z_ab_ > 1.96, then the mediated effect is significant at p < 0.05.

**Bold:** denotes statistical significance at p < 0.05.

### Perceived risk as a mediator of associations between perceived safety and constrained behavior

Perceived risk was found to have statistically significant mediated effects on the following associations for girls: between perceived road safety and both types of constrained behavior, and between each of the following: perceived incivilities, perceived personal safety, victimization and defensive behavior. The proportion mediated ranged from 21.6% to 59.8%.

For boys, perceived risk had a non-significant mediated effect (with the proportion mediated being 17%) for the association between perceived road safety and defensive behavior. Large mediated effects (with the proportion mediated ranging from 42.2% to 78.8%) were found between victimization and both types of constrained behavior for boys, and between victimization and avoidance behavior for girls. However, these effects were not statistically significant.

## Discussion

This study is among the first to examine whether parental perception of risk mediates associations between perceived safety/victimization and parental restriction of their adolescents’ physical activity. The findings demonstrated that for girls only, perceived risk was a significant mediator of associations between perceived road safety and avoidance/defensive behavior, and between each of the following: perceived incivilities, perceived personal safety, victimization, and defensive behavior. Possible reasons for perceived risk being a significant mediator of associations between the above variables for girls, and not for boys, include evidence that boys and girls are socialized differently with regard to risk-taking behavior from an early age and that parents tend to be more protective of daughters than of sons [21].

On average, boys in our study were subject to lower levels of parental restriction than were girls. It is possible that perceived risk is a more salient consideration in what parents allow their daughters to do, in comparison to sons. Several studies have demonstrated that boys are granted increased autonomy at an earlier age than are girls [7,22,23]. Furthermore, an English study reported that parental restriction of independent mobility was more prevalent for adolescent girls than for boys due to fears of molestation or assault [7]. Similar concerns were also expressed by parents of girls in a New Zealand study [24].

The findings of this study may guide the design of interventions that aim to increase levels of perceived safety and reduce perceptions of risk. Clearly, there is no risk of pedestrian injury and reduced risk of unwelcome approaches by strangers among adolescents whose leisure time is spent within the confines of the home. In order to promote the local neighborhood as a safe venue for adolescents’ physical activity, it is necessary to reduce concerns about road safety, incivilities and lack of personal safety (as all were associated with constrained behavior), and to lower perceptions of risk. To achieve this, interventions may reduce perceived risk by altering the physical environment (for example by implementing traffic calming measures that make residential streets more conducive to physical activity among children and adolescents [18]), by having well-lit streets to promote perceptions of safety among adolescent girls in particular [25], and by reducing the presence of physical incivilities such as graffiti and litter that may heighten perceptions of crime [26].

Alternatively, interventions may focus on reducing perceptions of risk in the context of the existing environment, and our findings suggest that these should target adolescent girls and their parents. Our earlier research found that that having friends living nearby was positively associated with adolescent girls’ walking in their neighborhood [27]. Walking groups for adolescent girls may promote perceptions of safety and encourage social interaction, as well as providing an important source of physical activity for this target group who typically engage in low and declining levels of physical activity [3,28].

Some limitations of this study, including high levels of education and of employment among parents, may impact the generalizability of the findings. Most respondents were mothers who may perceive risk differently from fathers, as there is evidence that females, compared with males, are socialized to take less risk [21]. It is possible that the reported levels of victimization, perceived risk and constrained behavior in this study may not be typical for all urban areas, and that different results may have been found in rural areas. Future research should investigate associations between these variables in diverse settings including rural areas. Further, perceived risk may be examined from a broader perspective to include other issues that may cause parents to restrict their adolescents’ physical activity within their neighborhood. These issues include bullying [29] and substance abuse [30]. Investigating potential mediating effects of these and other social and intrapersonal variables may be important, particularly among boys. In addition, further research that explores pathways that connect victimization, perceived risk and constrained behavior is warranted – although very strong mediating effects (> 77%) of perceived risk were present for boys, these pathways were not statistically significant.

## Conclusions

While the analyses presented here are exploratory in nature, they demonstrate that associations between aspects of perceived safety/victimization and constrained behavior are complex. In particular, the findings emphasize the importance of measuring perceived risk when examining how prior victimization and concerns about road safety, incivilities and personal safety are associated with constrained behavior. Because there is evidence that parents encourage independence among sons while promoting cautious behavior among daughters [21], it is imperative that environmental and/or social interventions aim to increase actual and perceived safety, and reduce perceptions of risk in safe neighborhoods so that adolescent girls in particular may be more physically active in their local neighborhoods.

## Competing interests

The authors declare that they have no competing interests.

## Authors’ contributions

AC was responsible for the overall conception and design of this manuscript, development of survey measures, statistical analysis and interpretation of data. AT was responsible for data acquisition, and contributed along with KH and DC to drafting and critical revision of the manuscript. All authors read and approved the final manuscript.
